# 

**Published:** 1898-05

**Authors:** 


### CONTENTS.

ORIGINAL COMMUNICATIONS—

Empyema of the Accessory Sinuses of the Nose. By Frank M. Hanger, M.D., Staunton, Va ..... 145
Mentally Deficient Children. By Katherine Collins, M.D., Atlanta, Ga ..... 155
Mastoid Inflammation with a Report of Two Interesting Cases. By T. E. Mitchell, M.D., Columbus, Ga ..... 164
Hemorrhage Following Removal of the Third Tonsil. By Ross P. Cox, M.D., Rome, Ga ..... 170
A Case of Presumable Hermaphrodism. By Edward Nicholas Liell, Jacksonville, Fla ..... 174

SOCIETY REPORTS—

Medical Association of Georgia ..... 177

EDITORIAL—

The Atlanta Quarantine Convention ..... 193
The Georgia Medical Association ..... 195

MEDICAL ITEMS ..... 197
BOOK REVIEWS ..... 203

SELECTIONS AND ABSTRACTS—

Skin Diseases and Syphilis ..... 207
Miscellaneous ..... 209

MEDICAL ITEMS—Continued ..... x, xi, xii, xiii, xiv, xvi, xvii
ADVERTISERS' INDEX TO ADVERTISEMENTS ..... xviii
CLASSIFIED INDEX TO ADVERTISEMENTS ..... xix

